# Supplementary material for: Pelagic larval duration, growth rate, and population genetic structure of the tidepool snake moray Uropterygius micropterus around the southern Ryukyu Islands, Taiwan, and the central Philippines
Source: PeerJ. 2018 May 9;6:e4741. doi: 10.7717/peerj.4741 (PMC5949063; doi:10.7717/peerj.4741)
Supplement: Table S1 [file peerj-06-4741-s001.docx]

| Eel groups | <45 days  (n = 38) | 45-60 days  (n = 109) | >60 days  (n = 37) |
| --- | --- | --- | --- |
| <45 days |  | 0.32 | 0.42 |
| 45-60 days | 0.0015 |  | 0.33 |
| >60 days | -0.0006 | 0.0012 |  |
