# Supplementary figures and images for: Pelagic larval duration, growth rate, and population genetic structure of the tidepool snake moray Uropterygius micropterus around the southern Ryukyu Islands, Taiwan, and the central Philippines
Source: PeerJ. 2018 May 9;6:e4741. doi: 10.7717/peerj.4741 (PMC5949063; doi:10.7717/peerj.4741)

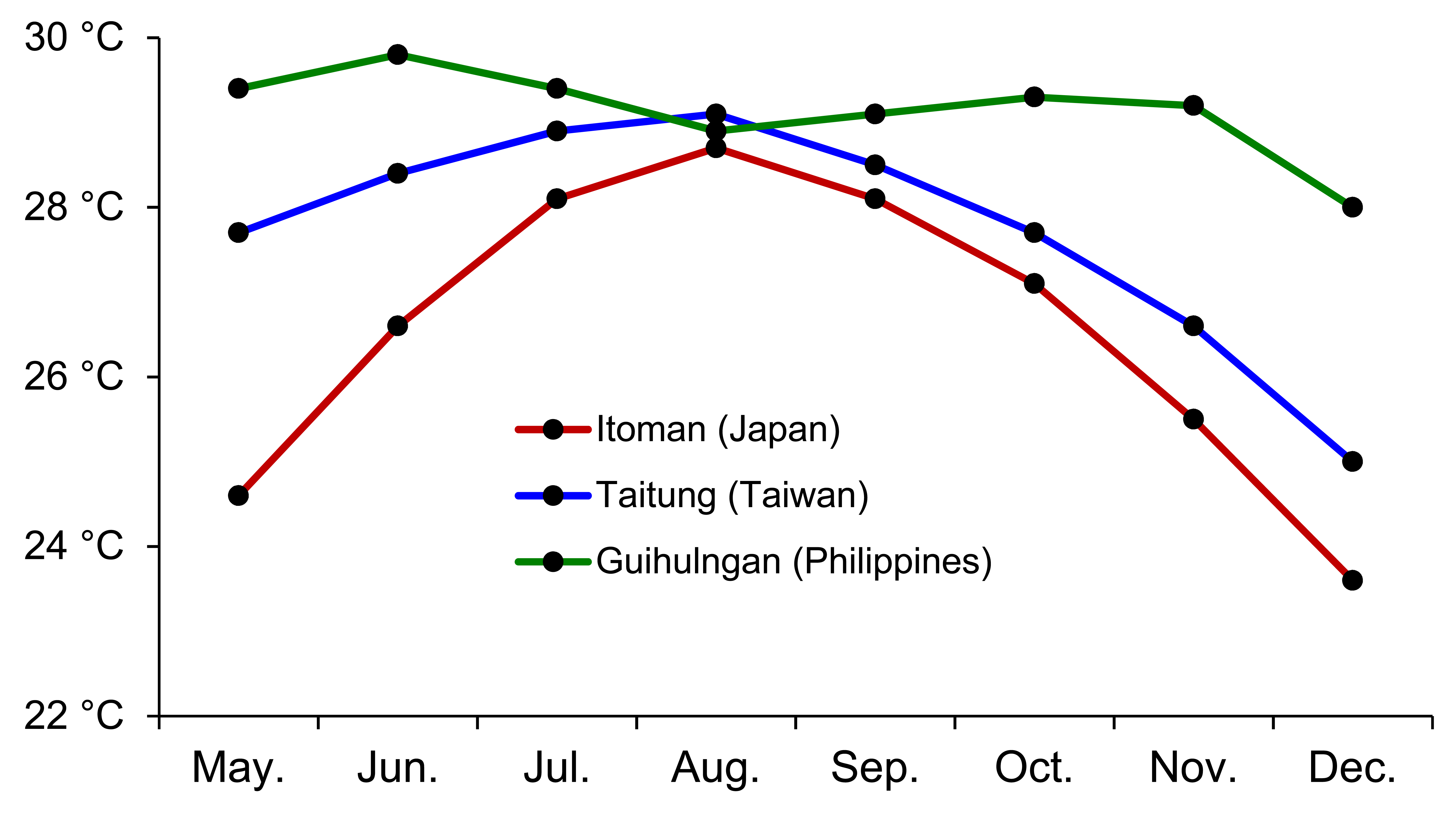

Supplement: Figure S1 — The time period that includes the spawning season and pelagic leptocephalus stage of Uropterygius micropterus. Data were obtained from the Global Sea Temperature website (http://www.seatemperature.org/). [file peerj-06-4741-s002.png]

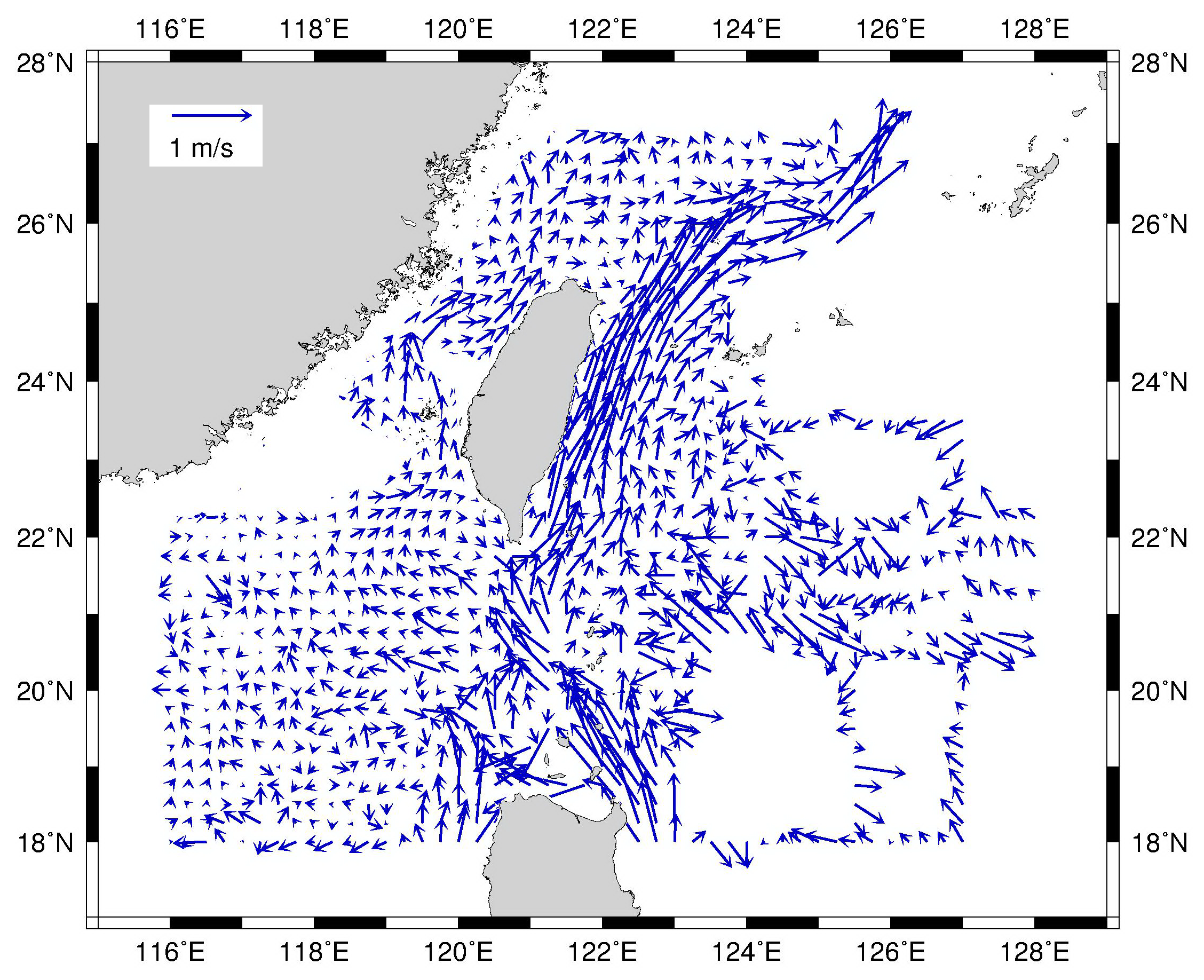

Supplement: Figure S2 — Data were obtained from the Ocean Data Bank of the Ministry of Science and Technology, Republic of China (http://www.odb.ntu.edu.tw/). [file peerj-06-4741-s003.png]
